# Supplementary material for: Anisotropic leaky-like perturbation with subwavelength gratings enables zero crosstalk
Source: Light Sci Appl. 2023 Jun 2;12:135. doi: 10.1038/s41377-023-01184-5 (PMC10238401; doi:10.1038/s41377-023-01184-5)
Supplement: Supplementary file 1 — Supplementary Information [file 41377_2023_1184_MOESM1_ESM.docx]

**Supplementary Information for**

**Anisotropic leaky-like perturbation with subwavelength gratings
enables zero crosstalk**

**Md Faiyaz Kabir^1^, Md Borhan Mia^1^, Ishtiaque Ahmed^2^, Nafiz Jaidye^2^, Syed Z. Ahmed^1^, and Sangsik Kim^1,2,3,*^**

^1^*Department of Electrical and Computer Engineering, Texas Tech University, Lubbock, Texas 79409, USA*

^2^*Department of Physics and Astronomy, Texas Tech University, Lubbock, Texas 79409, USA*

^3^*School of Electrical Engineering, Korea Advanced Institute of Science and Technology, Daejeon 34141, South Korea*

**Corresponding author:* [*sangsik.kim@kaist.ac.kr*](mailto:sangsik.kim@kaist.ac.kr)


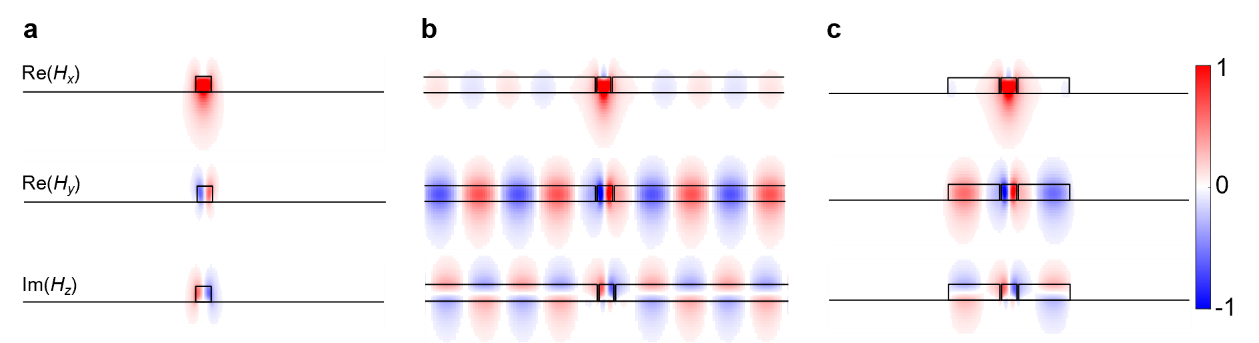


**Fig. S1. Magnetic field profiles of the strip, infinite subwavelength grating (infinite-SWG), and finite-SWG waveguides.** **a-c,** Magnetic field profiles of the fundamental TM modes (TM_0_) in each waveguide scheme: **a,** strip, **b,** infinite-SWG, and **c,** finite-SWG. From top to bottom, Re[*H_x_*], Re[*H_y_*], and Im[*H_z_*] of TM_0_ mode are plotted. The geometric parameters are the same as in Fig. 2 of the main manuscript.


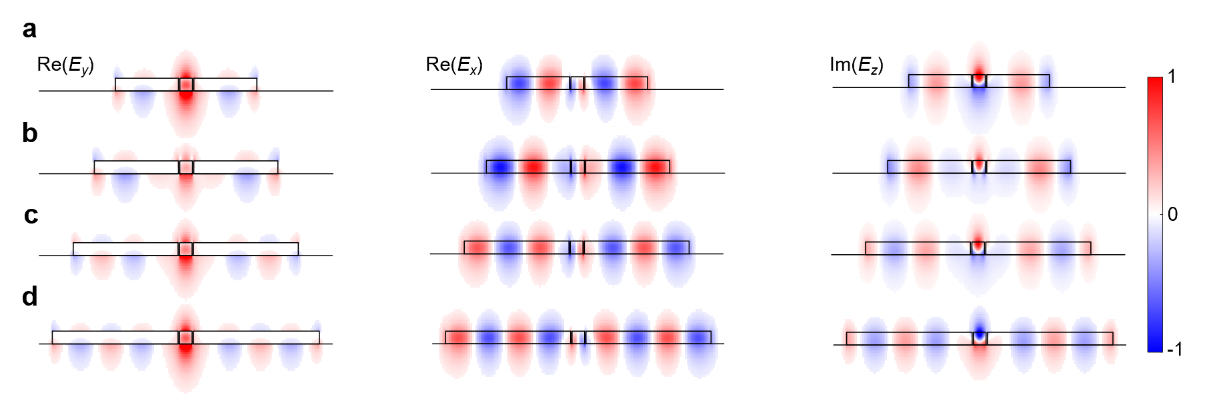


**Fig. S2. Mode profiles with *w*_swg_ variation for finite-SWG waveguides.** **a-d,** Mode profiles of fundamental TM modes (TM_0_) for different SWG widths: **a,** *w*_swg_ = 3 μm, **b,** *w*_swg_ = 4 μm, **c,** *w*_swg_ = 5 μm, and **d,** *w*_swg_ = 6 μm. From left to right, Re[*E_y_*], Re[*E_x_*], and Im[*E_z_*] are plotted. The oscillations of the anisotropic leaky-like SWG mode can be controlled by changing the SWG width, as seen from the different radiation patterns. The other geometric parameters are the same as in Fig. 2 of the main manuscript.


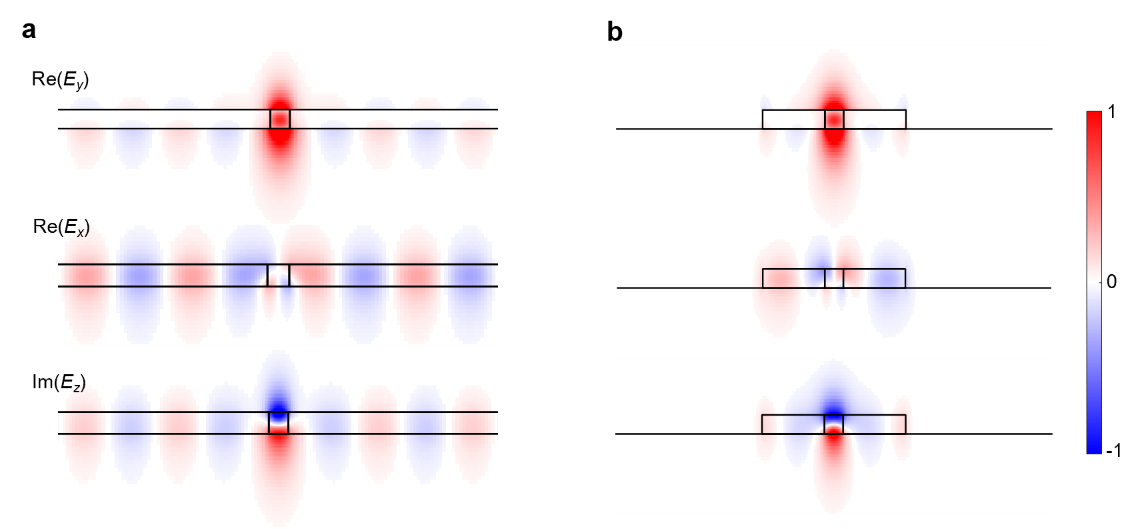


**Fig. S3. Modal properties of the infinite subwavelength grating (infinite-SWG) and finite-SWG waveguides without the gap between the core and the cladding.** **a, b,** Mode profiles of fundamental TM modes (TM_0_) in (**a**) infinite-SWG and (**b**) finite-SWG. From top to bottom, Re[*E_y_*], Re[*E_x_*], and Im[*E_z_*] are plotted. The mode profiles exhibit (**a**) a leaky mode for the infinite-SWG and (**b**) a hybrid mode with an oscillation for the finite-SWG, even without the gap between core and SWG cladding. The geometric parameters are the same as in Fig. 2.


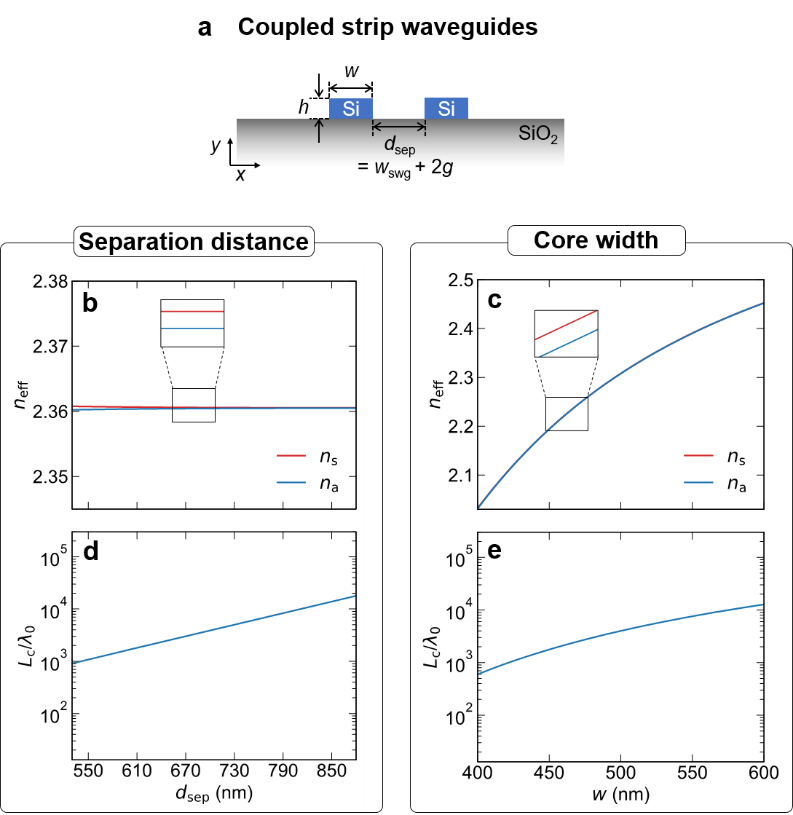


**Fig. S4. Modal simulation results of TE_0_ mode for coupled strip waveguides. a,** Schematic of the coupled strip waveguides. **b, c,** Numerically simulated effective indices of the coupled symmetric (*n*_s_, red) and anti-symmetric (*n*_a_, blue) TE_0_ modes for strip waveguides with variation in **b,** separation distance *d*_sep_, and **c**, core width *w*, and, **d, e**, their corresponding normalized coupling lengths *L*_c_/*λ*_0_. Due to the large confinement of the TE_0_ modes, the coupling length is significantly higher than the TM_0_ modes (Fig. 3e) for the same separation distance *d*_sep_ = *w*_swg_ + 2*g*.


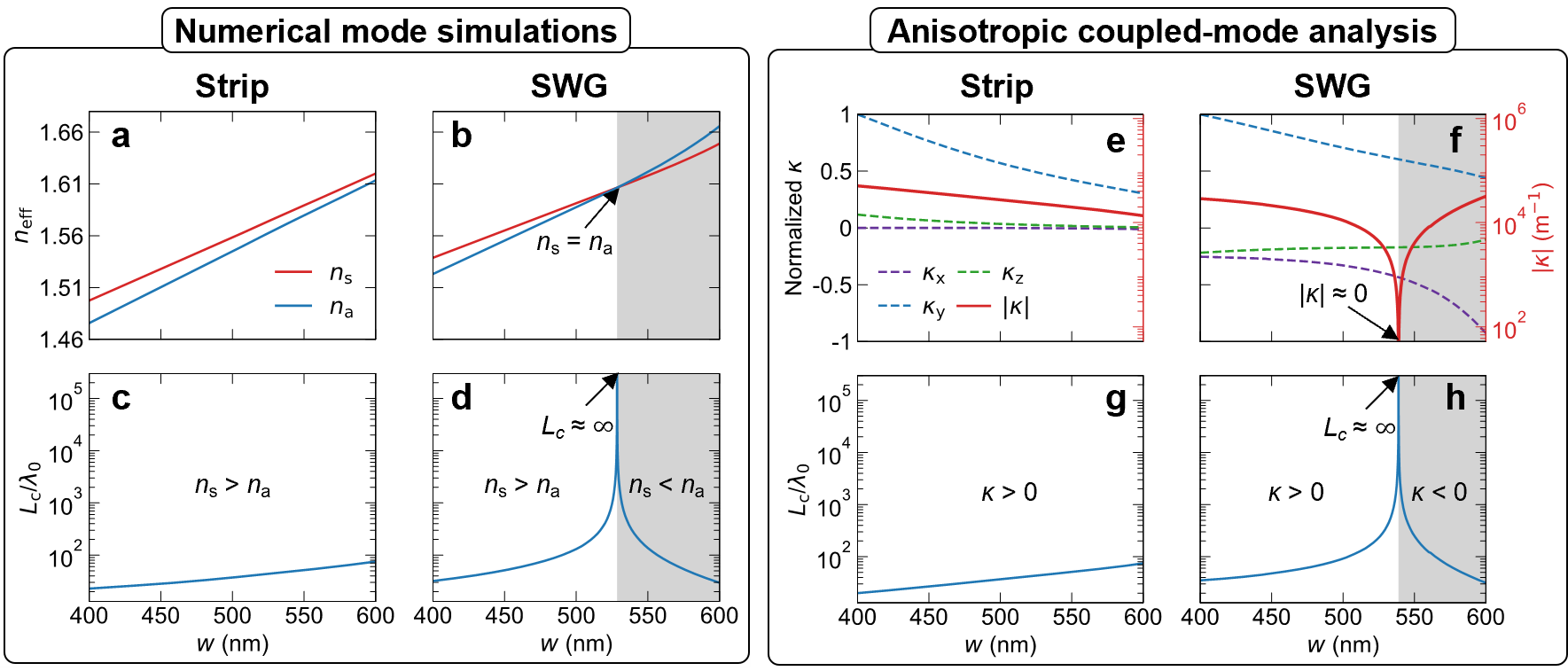


**Fig. S5.** **Coupling length vs. core width *w* variation in TM_0_ mode with coupled SWG waveguides.** **a, b,** Numerically simulated effective indices of the coupled symmetric (*n*_s_, red) and anti-symmetric (*n*_a_, blue) TM_0_ modes for **a**, strip and **b**, SWG waveguides, and **c, d**, their corresponding normalized coupling lengths *L*_c_/*λ*_0_. All the parameters and simulations are similar to Fig. 3 but as a function of the core width *w*. **e, f,** Normalized coupling coefficients *κ_x_* (purple dashed), *κ_y_* (blue dashed), *κ_z_* (green dashed), and the total coupling coefficient |*κ*|=|*κ_x_* + *κ_y_* + *κ_z_*| (red solid). **g, h,** Corresponding *L*_c_/*λ*_0_ for the coupled strip and SWG waveguides, respectively. The grey-shaded areas represent the non-trivial coupling region, where (**b, d**) *n*_a_ > *n*_s_ and (**f, h**) *κ* < 0**.** The free-space wavelength is *λ*_0_ = 1550 nm, and the other parameters are *h* = 220 nm, *w*_swg_ = 620 nm, and *g* = 65 nm.


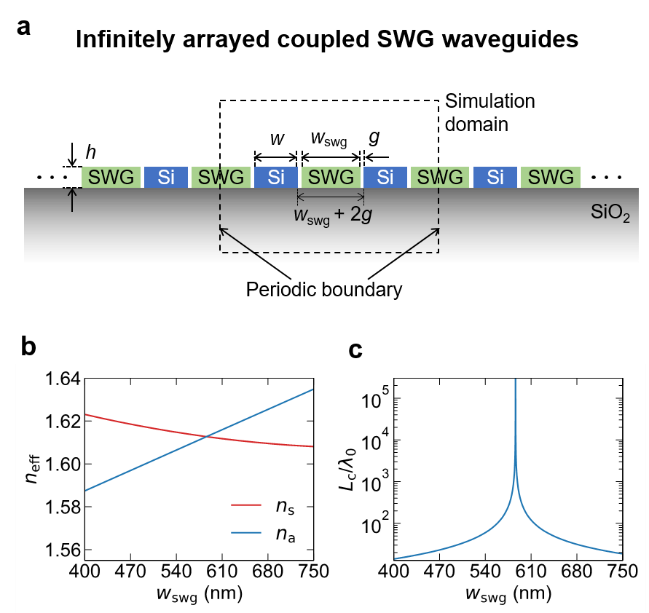


**Fig. S6.** Zero crosstalk in multiple SWG waveguides array. **a**, Schematic of infinitely-arrayed coupled SWG waveguides with periodic boundaries. All parameters are the same as in Fig. 3 of the main manuscript, except for the lateral boundary conditions. **b,** Numerically simulated effective indices of the coupled symmetric (*n*_s_, red) and anti-symmetric (*n*_a_, blue) TM_0_ modes, and, **c**, their corresponding normalized coupling lengths *L*_c_/λ_0_. This clearly show the idea shown in the main manuscript also can be applied to multiple waveguides array.


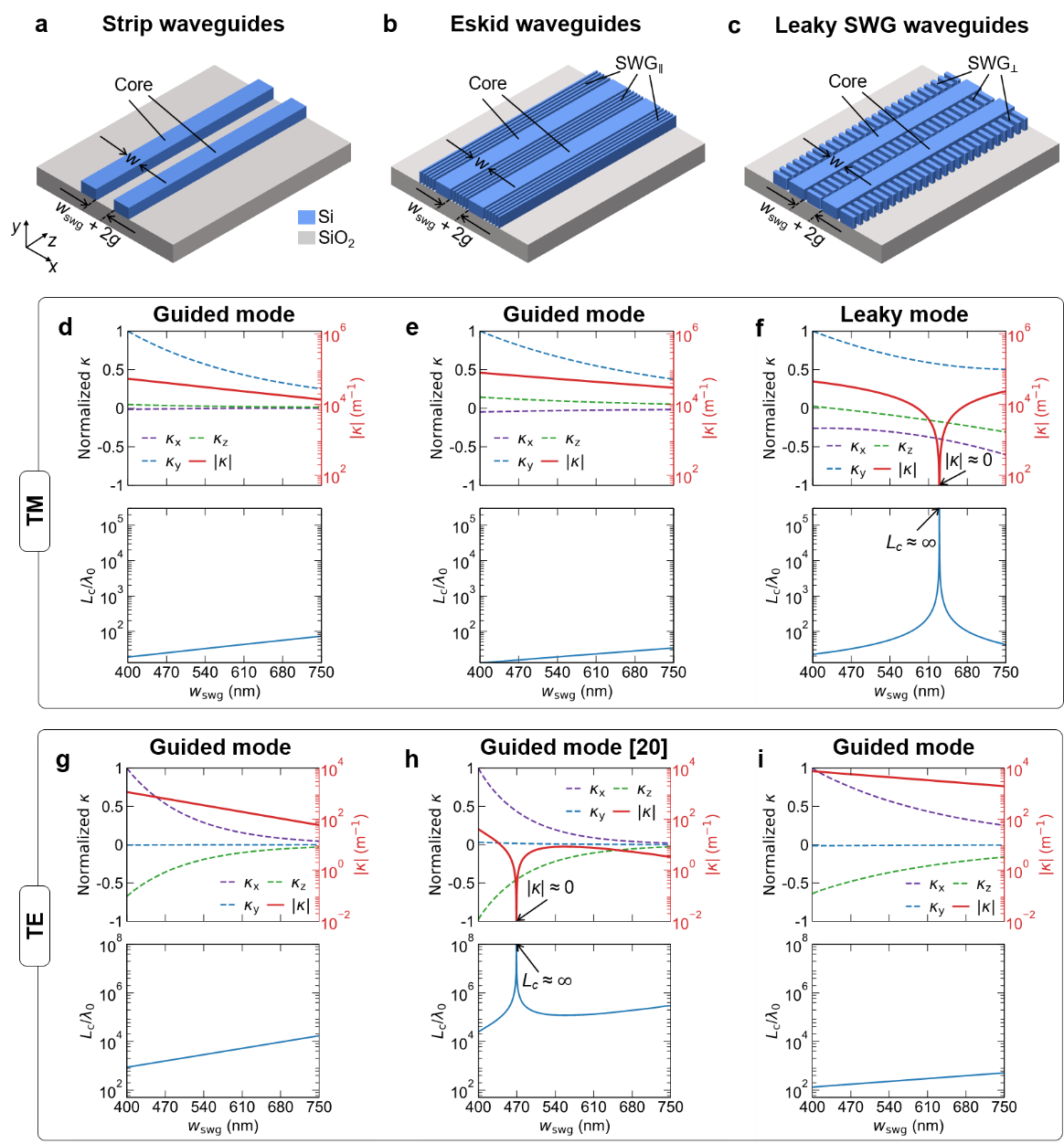


**Fig. S7. Comparison of dielectric perturbations and coupling lengths in different waveguide schemes for TE and TM modes. a-c**, Schematics of coupled **(a)** strip, **(b)** eskid (SWG_∥_), and **(c)** leaky SWG (SWG_⊥_) waveguides. **d-i,** Normalized coupling coefficients *κ_x_* (purple dashed), *κ_y_* (blue dashed), *κ_z_* (green dashed), and the total coupling coefficient |*κ*| = |*κ_x_* + *κ_y_* + *κ_z_*| (red solid) as a function of SWG width *w*_swg_, and the corresponding normalized coupling lengths *L*_c_/λ_0_ (blue solid) for **(d-f)** TM and **(g-i)** TE polarizations. The results are plotted for coupled **(d, g)** strip, **(e, h)** eskid, and **(f, i)** leaky SWG waveguides. The effective medium theory (EMT) is used to model the SWG_∥_ ($\varepsilon_{x}=\varepsilon_{\perp}$ and $\varepsilon_{y}=\varepsilon_{z}=\varepsilon_{||}$ ) and SWG_⊥_ ($\varepsilon_{x}=\varepsilon_{y}=\varepsilon_{||}$ and $\varepsilon_{z}=\varepsilon_{\perp}$ ) in **b** and **c**, respectively. Except for the leaky-like SWG mode in **f**, all the coupling coefficient components *κ_x_*, *κ_y_*, and *κ_z_* decrease as the separation distance (*w*_swg_ + 2*g*) increases due to exponentially decaying evanescent coupling. In contrast, for a leaky-like SWG mode in **f**, the *κ_x_* and *κ_z_* components show a non-conventional trend by increasing with *w*_swg_, canceling out the *κ_y_* component. The oscillating field components *E_x_* and *E_z_* in the anisotropic SWG enabled this non-conventional dielectric perturbation behavior. All parameters are kept the same as in Fig. 3 of the main manuscript.


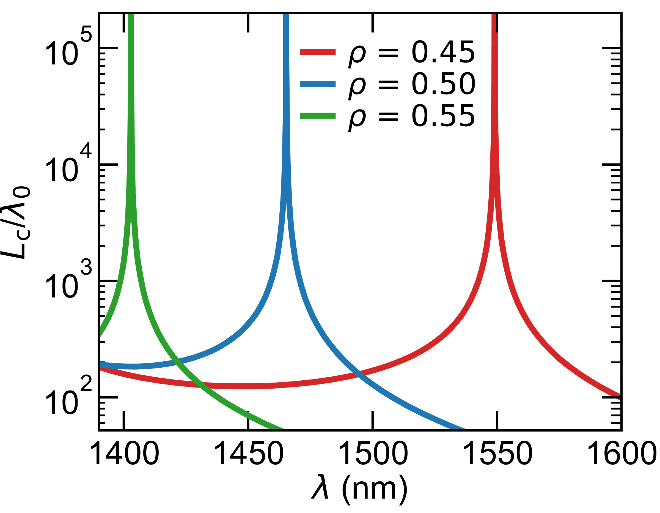


**Fig. S8. Effect of filling fraction on zero crosstalk wavelength.** Normalized coupling length *L*_c_/λ_0_ spectra for coupled leaky-SWG waveguides with different filling fractions *ρ* = 0.45 (red), 0.50 (blue), and 0.55 (green). Zero crosstalk (or infinite coupling length) wavelength shifts by Δλ_zero_≈ ±73 nm for filling fraction variation of ∆*ρ*=±0.05. All other parameters are the same as in Fig. 3b.


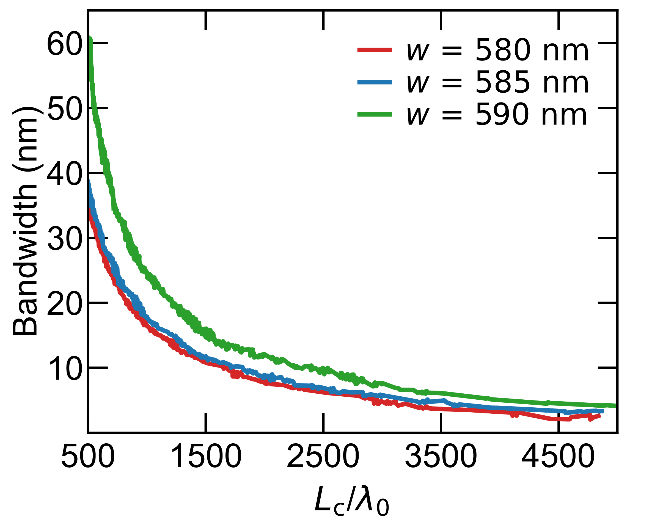


**Fig. S9. Bandwidth analysis of the coupled SWG mode with anisotropic perturbation.** The bandwidth of the experimental results in Fig. 4g is plotted as a function of normalized coupling length *L*_c_/λ_0_. The core width is *w* = 580 nm (red), 585 nm (blue), and 590 nm (green). A trade-off is observed between *L*_c_/λ_0_ and bandwidth, with a bandwidth of ≈20.1±3.0 nm for *L*_c_/λ_0_ > 1000 waves (corresponding to crosstalk suppression of ≈ 30 dB).
